# Supplementary material for: Raman Spectroscopy and Imaging Studies of Human Digestive Tract Cells and Tissues—Impact of Vitamin C and E Supplementation
Source: Molecules. 2022 Dec 24;28(1):137. doi: 10.3390/molecules28010137 (PMC9822473; doi:10.3390/molecules28010137)
Supplement: Supplementary file 1 [file molecules-28-00137-s001.zip › molecules-1990927-supplementary.pdf]

Supplementary Materials

Results for CCD-18 Co cells treated with tBHP, and vitamin C in concentration 25 µM and 50 µM of upon ROS generation are presented below. Raman bands in spectra presented in Figs. S1-S2 on panels D are ascribed to nucleic acids, amino acids, proteins and lipids, thereby providing chemical characterization of CCD-18 Co.

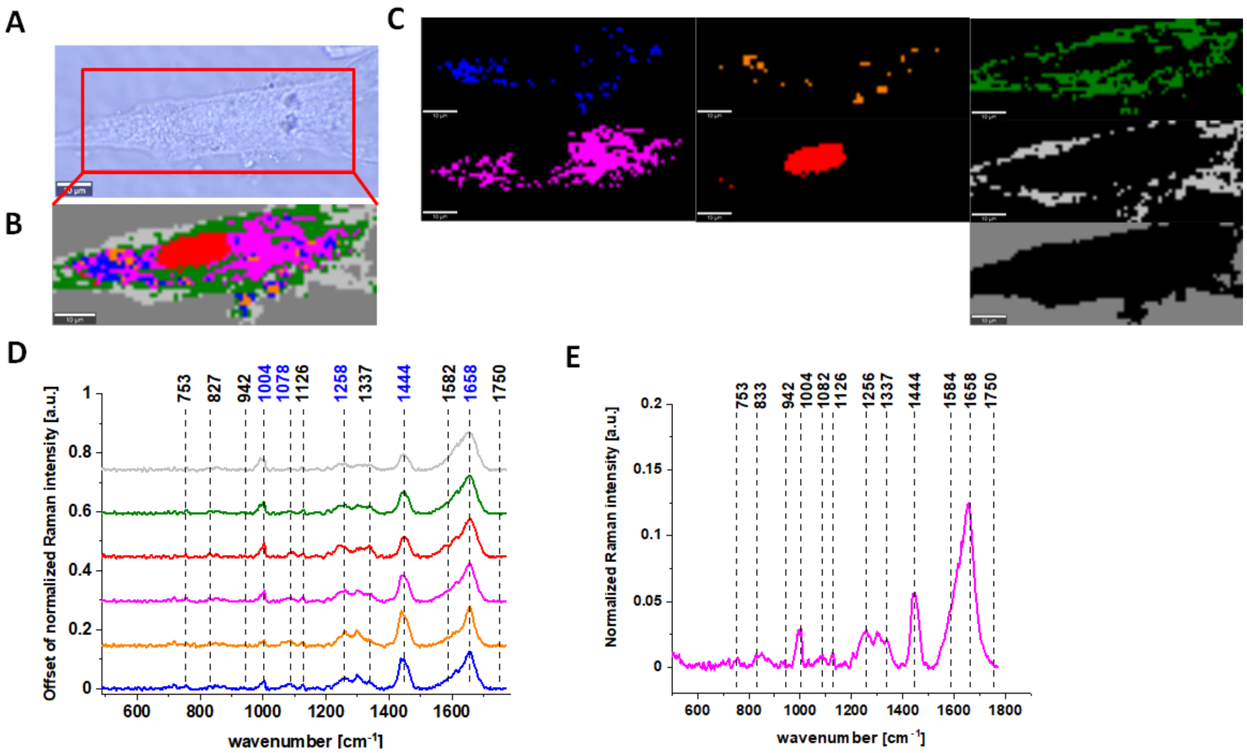

Fig. S1: The microscopy image of normal colon CCD-18 Co cell subjected to vitamin C (25 µM) and tBHP (50 µM) treatment (A), Raman image constructed based on Cluster Analysis (CA) method (B), Raman images of all clusters identified by CA assigned to: nucleus (red), mitochondria (magenta), lipid-rich regions: Lipid Droplets, lysosomes, endoplasmic retikulum (blue, orange), cytoplasm (green), membrane (light grey), and cell environment (dark grey) (C), average Raman spectra typical for all clusters identified by CA in a fingerprint region (D), average Raman spectrum for the whole cell in a fingerprint region (E), cells measured in PBS, excitation wavelength 532 nm.

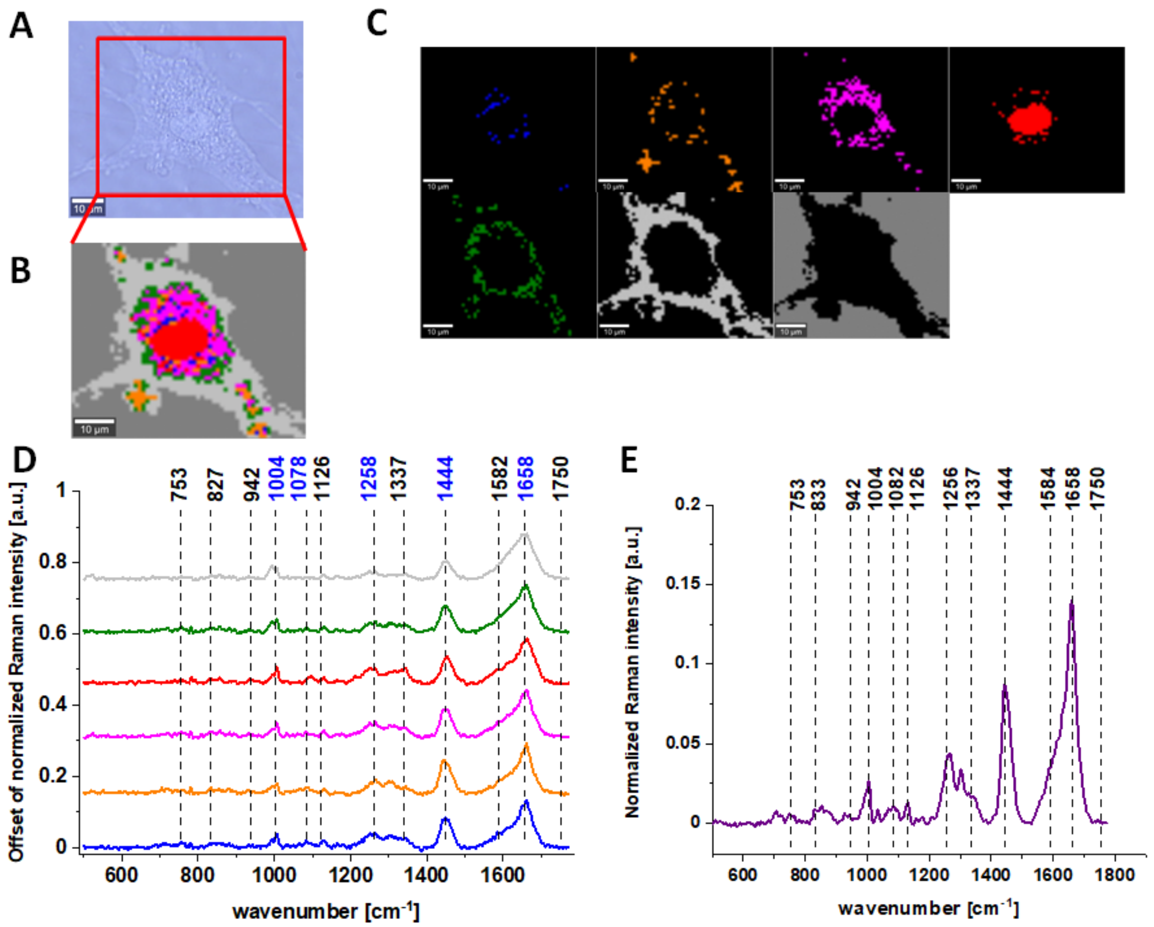

Fig. S2: The microscopy image of normal colon CCD-18 Co cells subjected to vitamin C (50 µM) and tBHP (50 µM) treatment (A), Raman image constructed based on Cluster Analysis (CA) method (B), Raman images of all clusters identified by CA assigned to: nucleus (red), mitochondria (magenta), lipid-rich regions: Lipid Droplets, lysosomes, endoplasmic retikulum (blue, orange), cytoplasm (green), membrane (light grey), and cell environment (dark grey) (C), average Raman spectra typical for all clusters identified by CA in a fingerprint region (D), average Raman spectrum for the whole cell in a fingerprint region (E), cells measured in PBS, excitation wavelength 532 nm.

Results for HTB-135 cancer gastric cells treated with 25µM and 25µM of vitamin E, are presented below. Signals in spectra presented in Figs. S4-S6 are ascribed to nucleic acids, amino acids, proteins and/or lipids, thereby providing biochemical characterization of HTB-135.

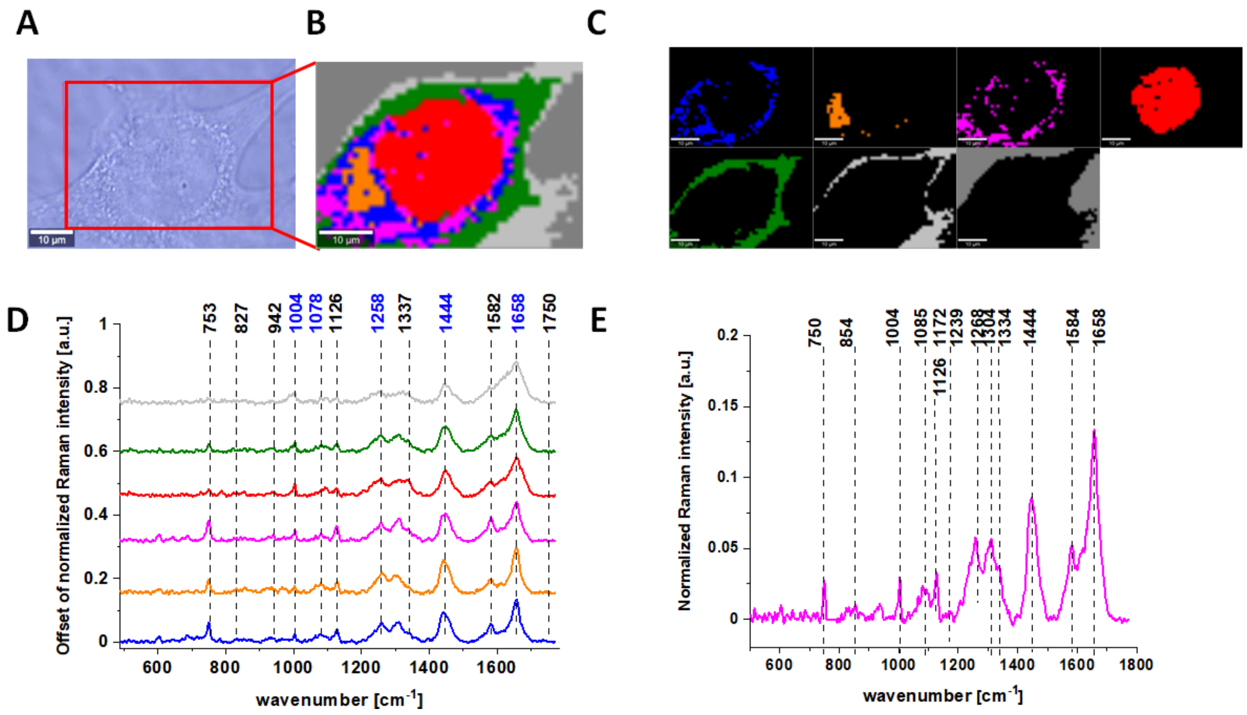

Fig. S3: The microscopy image of human cancer gastric HTB-135 cell exposed to 25 µM vitamin E (A), Raman image constructed based on Cluster Analysis (CA) method (B), Raman images of all clusters identified by CA assigned to: nucleus (red), mitochondria (magenta), lipid-rich regions: Lipid Droplets, lysosomes, endoplasmic retikulum (blue, orange), cytoplasm (green), membrane (light grey), and cell environment (dark grey) (C), average Raman spectra typical for all clusters identified by CA in a fingerprint region (D), average Raman spectrum for the whole cell in a fingerprint region (E), cells measured in PBS, excitation wavelength 532 nm.

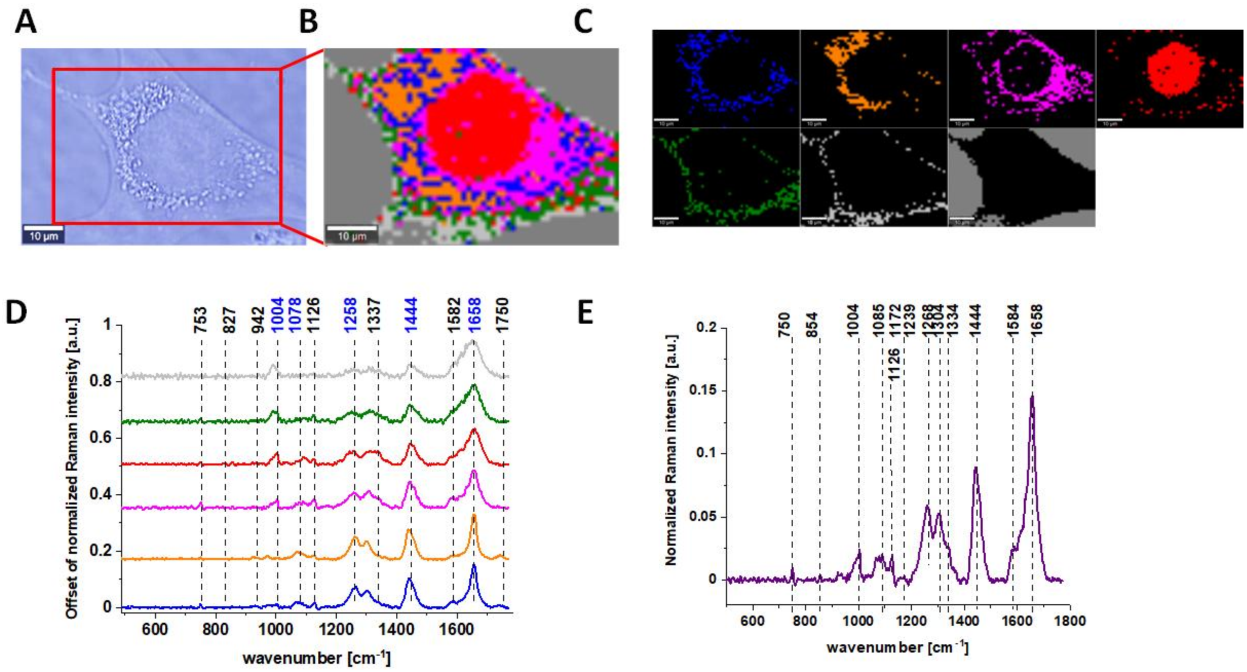

Fig. S4: The microscopy image of human cancer gastric HTB-135 cell exposed to 50 µM vitamin E (A), Raman image constructed based on Cluster Analysis (CA) method (B), Raman images of all clusters identified by CA assigned to: nucleus (red), mitochondria (magenta), lipid-rich regions: Lipid Droplets, lysosomes, endoplasmic retikulum (blue, orange), cytoplasm (green), membrane (light grey), and cell environment (dark grey) (C), average Raman spectra typical for all clusters identified by CA in a fingerprint region (D), average Raman spectrum for the whole cell in a fingerprint region (E), cells measured in PBS, excitation wavelength 532 nm.

Having reached this point, when the analysis based on Raman vibrational spectra for different normal human colon cells CCD-18 Co cells and cancerous human gastric (HTB-135) cells groups has been performed, we can comprehensively compare the results shown in Figures S5–S10.

Considering that CCD-18 Co human normal colon cells are basically composed of three types of macromolecules: proteins, nucleic acids and lipids, in order to explore characteristic changes under ROS conditions and for cells supplemented with vitamin C, the qualitative and quantitative comparison between paired bands assigned to those classes of compounds will be discussed according to the biological attribution of them. Figure S5-S7 shows an analysis of the influence of oxidative stress generating factor—tBHP and antioxidant in the form of vitamin C on analyzed cellular substructures identified in the cluster analysis. Band 1658 cm^{-1} describe the protein content in cells, amide III bands 1078 cm^{-1} reflect the amount of nucleic acids present in cells; bands 1444 cm^{-1} are characteristic for lipids. During the statistical data analysis the intensity of the peak at 1004 cm^{-1} was kept constant. We have also compared the results obtained for normal human colon cells with results typical for cancer cells CaCo-2.

Figure S5 shows the analysis based on the bands characteristic for nucleic acids. Figure below shows the results for all cell organelles.

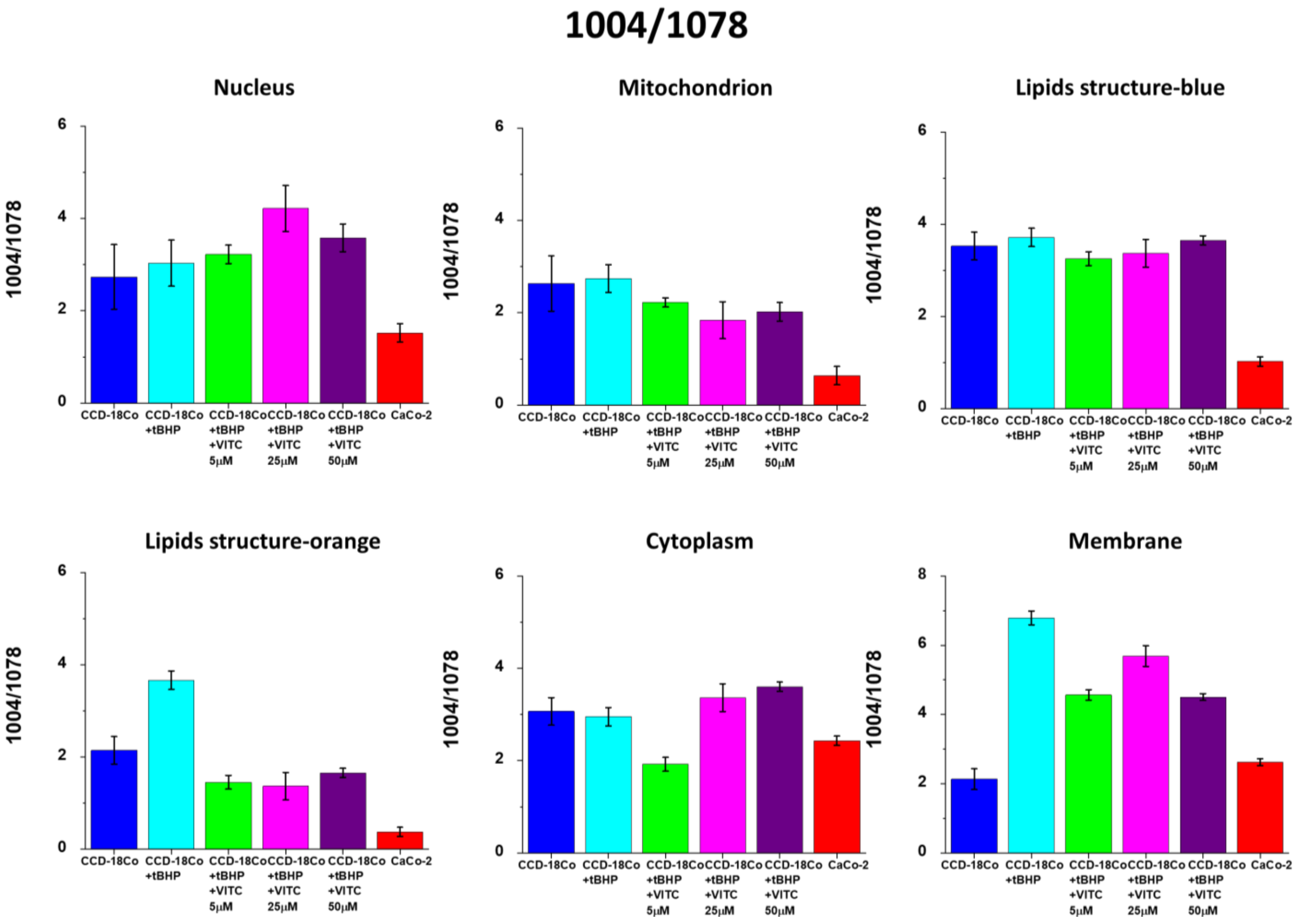

Fig. S5: Raman band intensity ratios for cellular substructures (nucleus, mitochondrion, lipids structure-blue, lipids structure-orange, cytoplasm, membrane) for Raman bands corresponding to nucleic acids for six groups of human colon cells: human normal colon cells - control group (labelled CCD-18-Co, blue), group supplemented with tBHP for 24 h (labelled CCD-18 Co+ tBHP, turquoise), group supplemented with tBHP and vitamin C 5μM (labelled CCD-18 Co+ tBHP + VITC 5μM, green), group supplemented with tBHP and vitamin C 25μM (labelled CCD-18 Co+ tBHP + VITC 25μM, magenta), group supplemented with tBHP and vitamin C 50μM (labelled CCD-18 Co+ tBHP + VITC 50μM, violet), human colon cancer cells CaCo-2 (labelled with CaCo-2, red).

As shown in Figure S5 for cells from human colon CCD-18 Co with a normal structure the addition of tBHP - an ROS generating agent caused a change in the values of the analyzed Raman band intensity ratios. The addition of tBHP resulted in a decrease in the intensity of bands typical for nucleic acids for all supplementations. Nucleic acids are much more stable than proteins and, when exposed to ROS, do not easily transform into a free radical state (damage is usually quickly repaired, damaged bases are "excised" and excreted from cells).

Figure S6 shows the analysis based on the band’s characteristic for lipids.

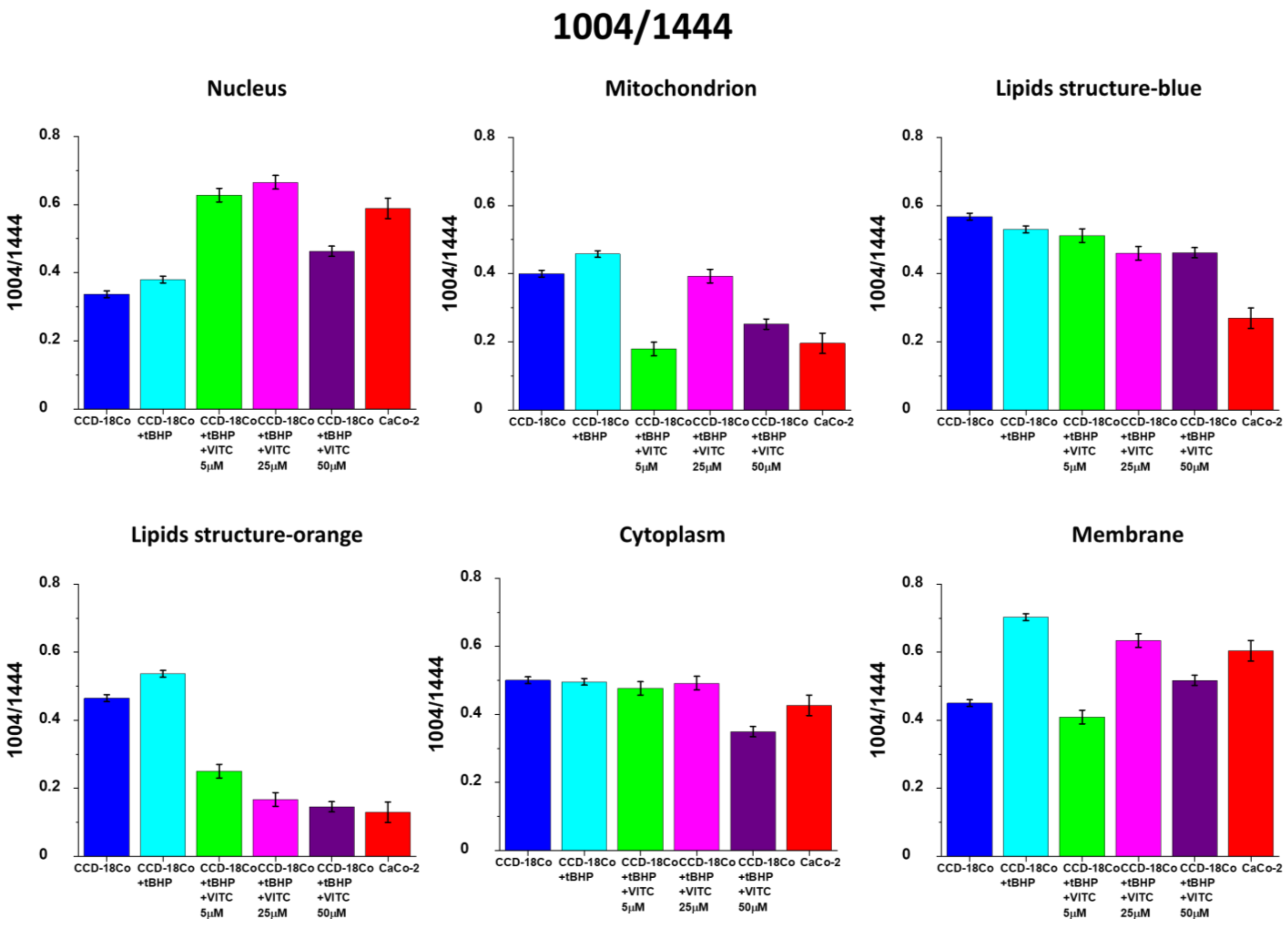

Fig. S6: Raman band intensity ratios for cellular substructures (nucleus, mitochondrion, lipids structure-blue, lipids structure-orange, cytoplasm, membrane) for Raman bands corresponding to lipids for six groups of human colon cells: human normal colon cells - control group (labelled CCD-18-Co, blue), group supplemented with tBHP for 24 h (labelled CCD-18 Co+ tBHP, turquoise), group supplemented with tBHP and vitamin C 5μM (labelled CCD-18 Co+ tBHP + VITC 5μM, green), group supplemented with tBHP and vitamin C 25μM (labelled CCD-18 Co+ tBHP + VITC 25μM, magenta), group supplemented with tBHP and vitamin C 50μM (labelled CCD-18 Co+ tBHP + VITC 50μM, violet), human colon cancer cells CaCo-2 (labelled with CaCo-2, red).

As can be seen in Figure S6 an agent generating reactive oxygen species - tBHP - contributes to the destruction of lipids through their oxidation. Lipid oxidation products can react with proteins and DNA, contributing to their further damage. The analysis of the Raman band intensity ratios 1004/1444 confirms the destructive effect of tBHP on lipids and the "protective" nature of vitamin C for all the variants of antioxidant supplementation used.

Figure S7 shows the analysis based on the band’s characteristic for proteins.

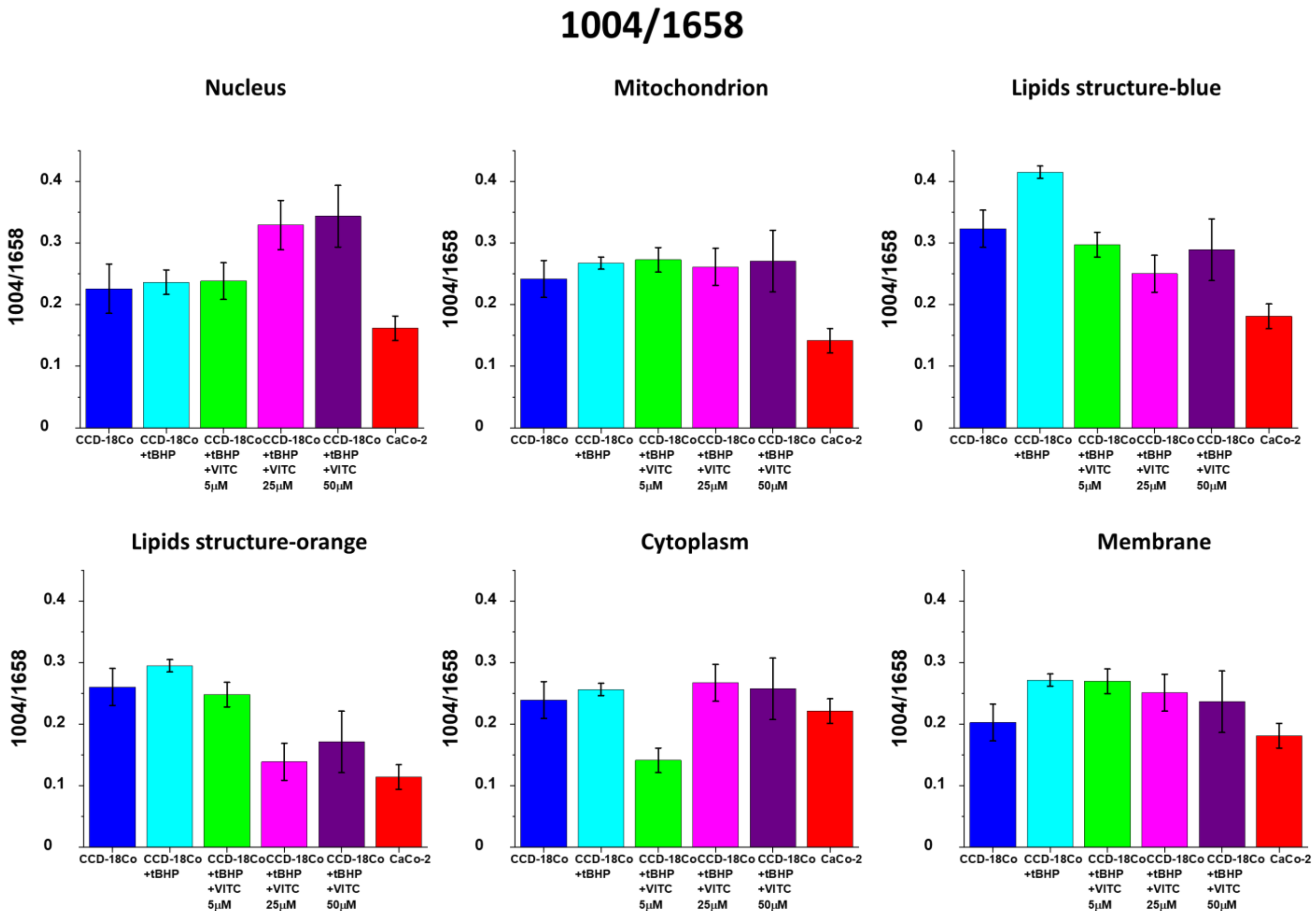

Fig. S7: Raman band intensity ratios for cellular substructures (nucleus, mitochondrion, lipids structure-blue, lipids structure-orange, cytoplasm, membrane) for Raman bands corresponding to proteins for six groups of human colon cells: human normal colon cells - control group (labelled CCD-18-Co, blue), group supplemented with tBHP for 24 h (labelled CCD-18 Co+ tBHP, turquoise), group supplemented with tBHP and vitamin C 5μM (labelled CCD-18 Co+ tBHP + VITC 5μM, green), group supplemented with tBHP and vitamin C 25μM (labelled CCD-18 Co+ tBHP + VITC 25μM, magenta), group supplemented with tBHP and vitamin C 50μM (labelled CCD-18 Co+ tBHP + VITC 50μM, violet), human colon cancer cells CaCo-2 (labelled with CaCo-2, red).

As shown in Figure S7, for normal human colon cells CCD-18 Co, the addition of tBHP - an ROS generating agent modulates content of proteins. As the intensity of the 1004 cm^{-1} band is practically constant after the addition of vitamin C compared to non-supplemented cells (which is confirmed in Figure S7), the effect of increasing the value of the ratio 1004/1658 for the CCD-18 Co + TBHP system results from a decrease in the intensity of the 1658 cm^{-1} band, and thus the modification of proteins after cell supplementation with a free radical generator. The results for supplementation showed a "protective" effect of the antioxidant in the form of vitamin C.

Considering that HTB-135 human gastric cancer cells are basically composed of three types of macromolecules: proteins, nucleic acids and lipids, to explore characteristic changes under supplementation with vitamin E, the qualitative and quantitative comparison between paired bands assigned to those classes of compounds will be discussed according to the biological attribution of them. Figure S8-S10 shows an analysis of the influence antioxidant in the form of vitamin E on analyzed cellular substructures identified in the cluster analysis. Band 1658 cm^{-1} describe the protein content in cells, amide III bands 1078 cm^{-1} reflect the amount of nucleic acids present in cells; bands

1444 cm^{-1} are characteristic for lipids. During the statistical data analysis, the intensity of the peak at 1004 cm^{-1} was kept constant. We have also compared the results obtained for human gastric cancer cells with results typical for human normal gastric cells CRL7869.

Figure S8 shows the analysis based on the band's characteristic for nucleic acids.

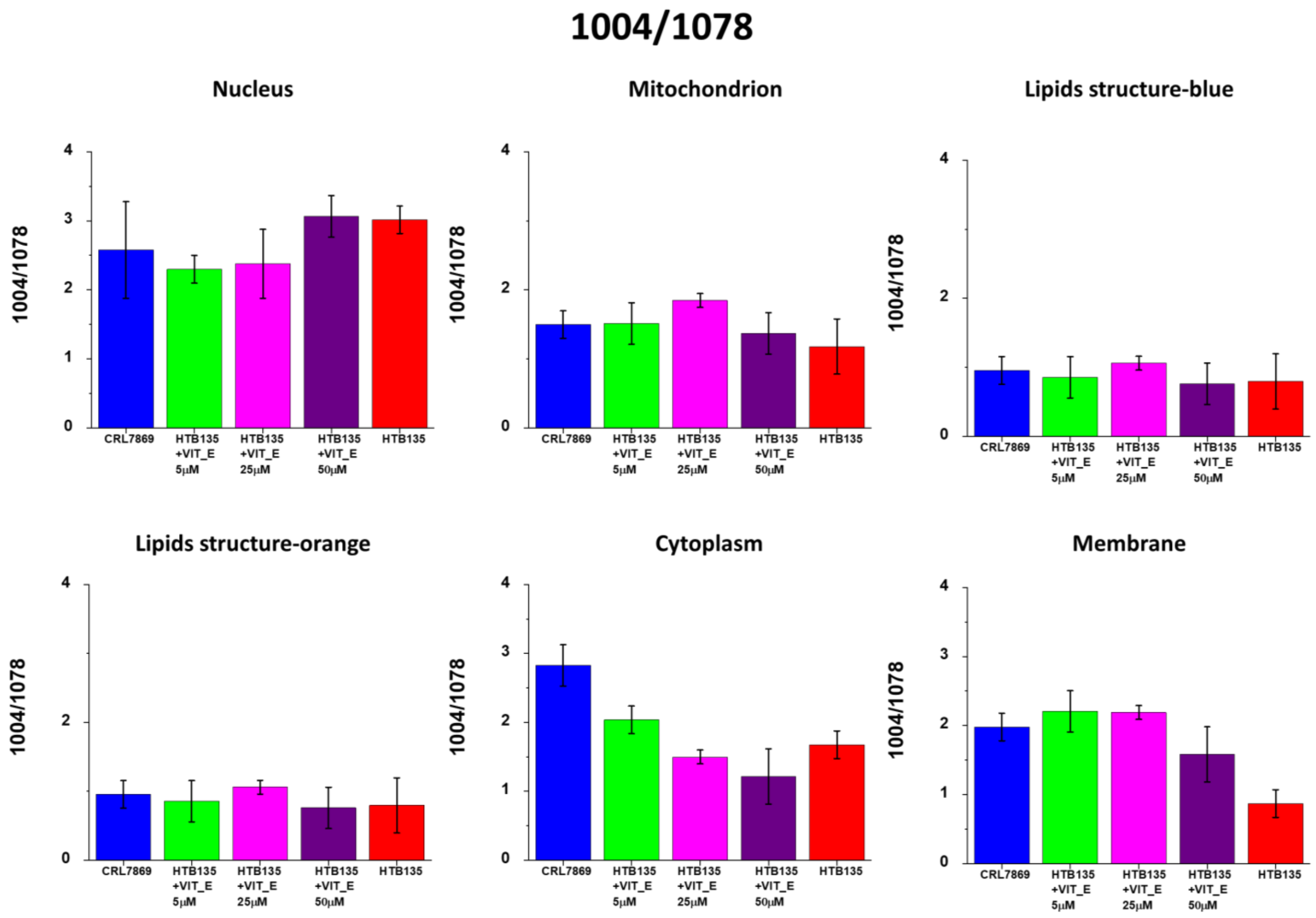

Fig. S8: Raman band intensity ratios for cellular substructures (nucleus, mitochondrion, lipids structure-blue, lipids structure-orange, cytoplasm, membrane) for Raman bands corresponding to nucleic acids for five groups of human gastric cells: human gastric cancer cells - control group (labelled HTB135, blue), group supplemented with vitamin E in concentration of 5μM (labelled HTB-135 + VIT_E 5μM, green), group supplemented with vitamin E in concentration of 25 μM (labelled HTB-135 + VIT_E 25 μM, magenta), group supplemented with vitamin E in concentration 50 μM (labelled HTB-135 + VIT_E 50 μM, violet), group of pure normal gastric cell (labelled CRL7869, blue). During the statistical data analysis, the intensity of the peak at 1004 cm^{-1} was kept constant.

As shown in the Figure S8, the addition of vitamin E affects the content of nucleic acids in gastric cancer cells. The most optimal and most effective vitamin E concentration is 25 μM. It causes the most beneficial changes in many organelles. However, in the most important structures containing genetic material, the higher dose (50 μM) of vitamin E is the appropriate one.

Figure S9 shows the analysis based on the band's characteristic for lipids.

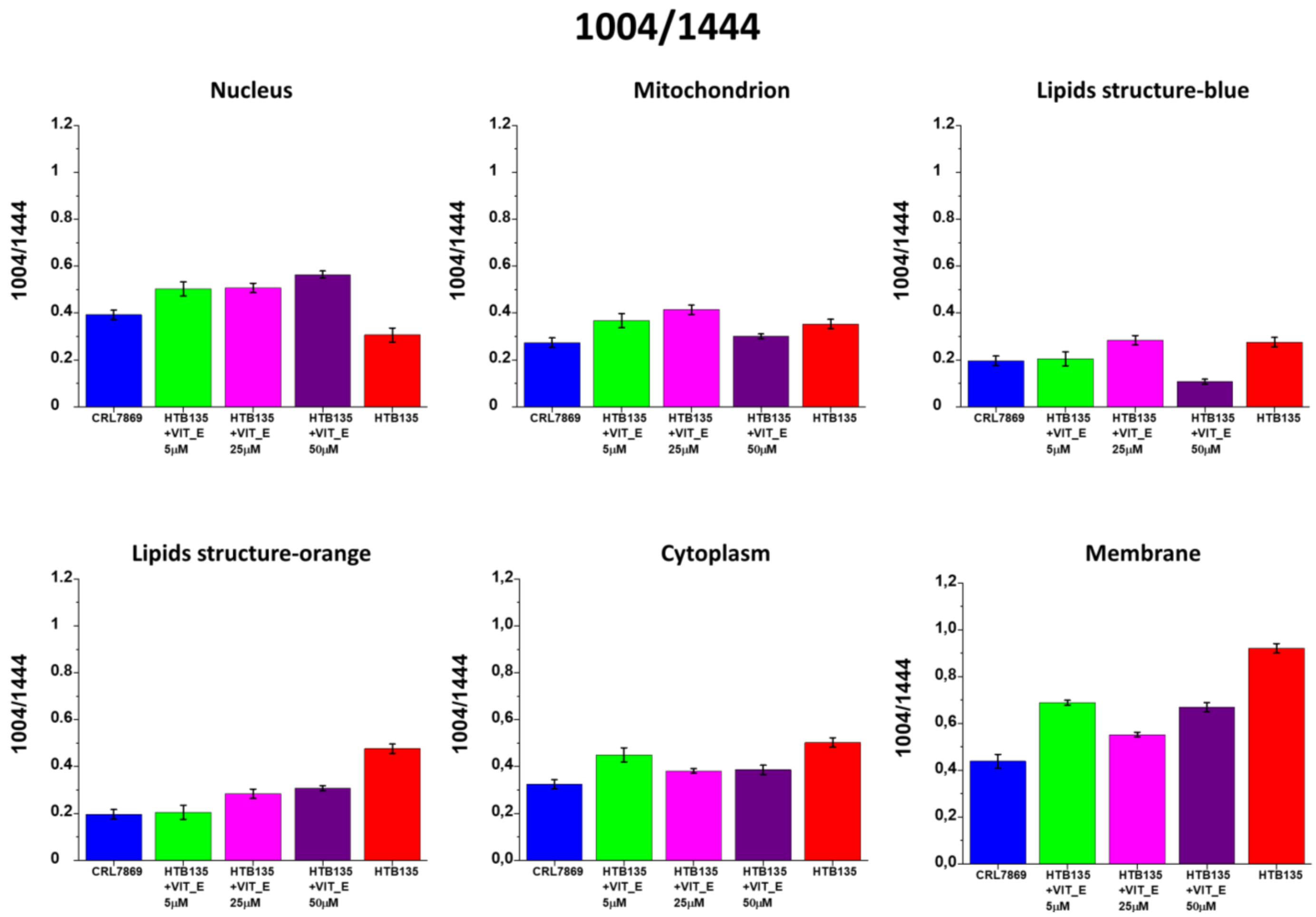

Fig. S9: Raman band intensity ratios for cellular substructures (nucleus, mitochondrion, lipids structure-blue, lipids structure-orange, cytoplasm, membrane) for Raman bands corresponding to lipids for five groups of human gastric cells: human gastric cancer cells - control group (labeled HTB135, blue), group supplemented with vitamin E in concentration of 5μM (labelled HTB-135 + VIT_E 5μM, green), group supplemented with vitamin E in concentration of 25 μM (labelled HTB-135 + VIT_E 25 μM, magenta), group supplemented with vitamin E in concentration 50 μM (labelled HTB-135 + VIT_E 50 μM, violet), group of pure normal gastric cell (labelled CRL7869, blue). During the statistical data analysis, the intensity of the peak at 1004 cm^{-1} was kept constant.

As can be seen in the picture, the addition of vitamin E also affects the lipid content in gastric cancer cells. The most optimal and most effective vitamin E concentration is 25 μM. The highest value of the frequency ratio presented for this concentration allows the conclusion that it shows the most "blocking" nature of the antioxidant. The effect is most visible in lipid structures. In the case of observation of structures such as the cell nucleus or the membrane, a slightly greater effect is caused by the vitamin E concentration of 50 μM.

Figure S10 shows the analysis based on the band's characteristic for proteins.

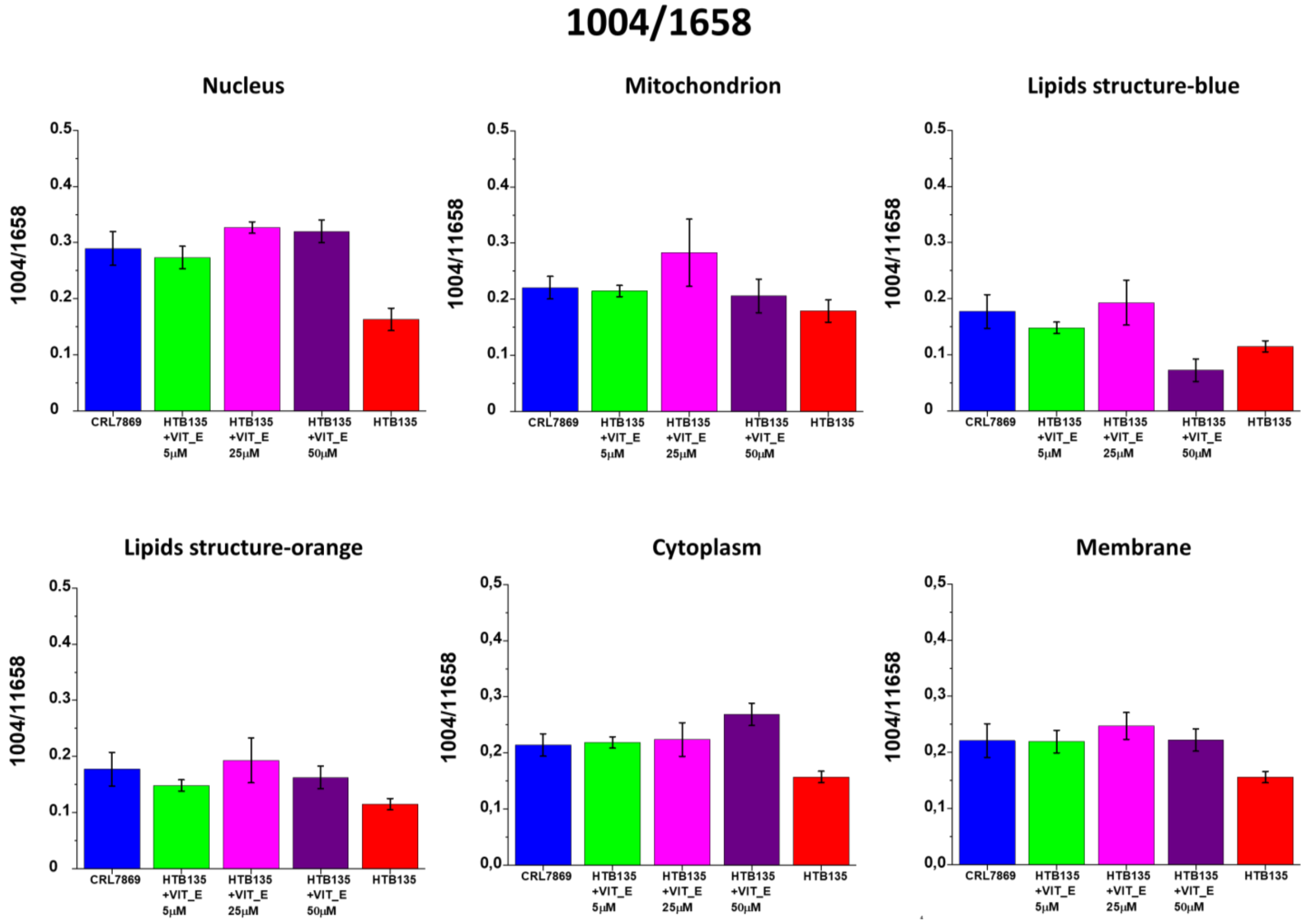

Fig. S10: Raman band intensity ratios for cellular substructures (nucleus, mitochondrion, lipids structure-blue, lipids structure-orange, cytoplasm, membrane) for Raman bands corresponding to lipids for five groups of human gastric cells: human gastric cancer cells - control group (labeled HTB135, blue), group supplemented with vitamin E in concentration of 5μM (labelled HTB-135 + VIT_E 5μM, green), group supplemented with vitamin E in concentration of 25 μM (labelled HTB-135 + VIT_E 25 μM, magenta), group supplemented with vitamin E in concentration 50 μM (labelled HTB-135 + VIT_E 50 μM, violet), group of pure normal gastric cell (labelled CRL7869, blue). During the statistical data analysis the intensity of the peak at 1004 cm^{-1} was kept constant.

As shown in Figures S8-S10, the effect of vitamin E on gastric cancer cells is significant. The addition of vitamin E to cells with a cancerous changed structure causes the inhibition of cancer progression, thus affecting all cellular functions. As can be seen from the presented graphs on Figures S8-S10, the most significant effect of the antioxidant addition is observed in the cytoplasm and lipid structures marked in blue. All the obtained results allow us to conclude that vitamin supplementation allows to reduce the risk of cancer and, in the case of its presence, to inhibit its growth.

To prove that biochemical changes can be tracking by Raman spectroscopy and imaging we have performed also PCA analysis based on Raman spectra. Figures S11 - S22 show results obtained for investigated human cancer cells (Caco-2) and normal (CCD-18 Co) and treated by ROS generating agent and supplemented with vitamin C cells of colon and stomach normal cells (CRL-7869) and cancer cells (HTB-135) supplemented with vitamin E. Moreover, PCA for cells show the scores 3D

plots in two different spatial projections. In Principle Component Analysis for cells from colon cell line we took cognisance of 6 groups and for cells from stomach cell line 5 groups.

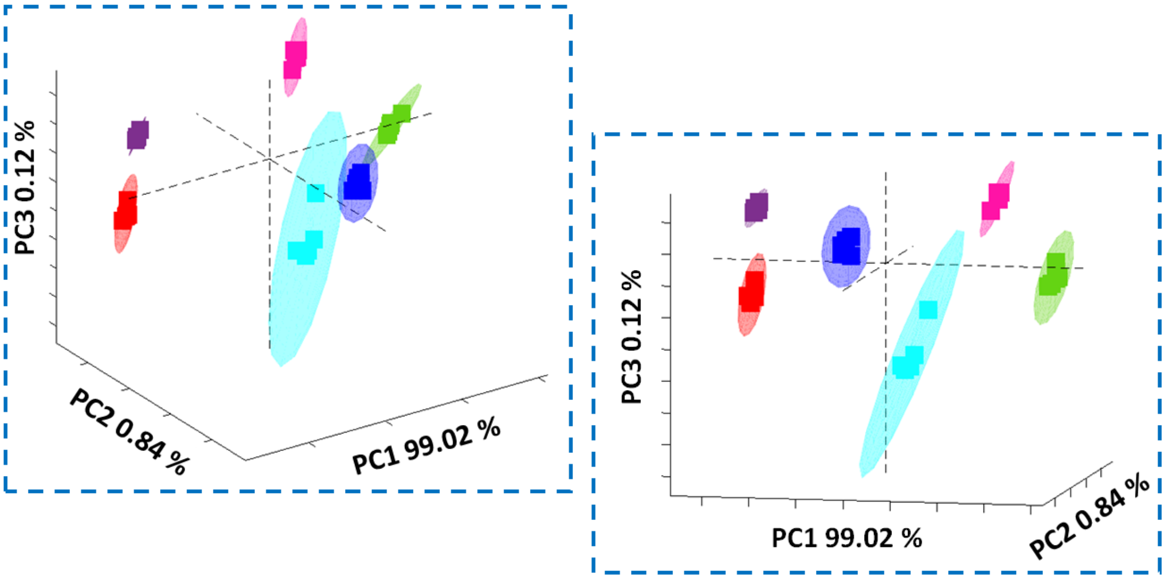

Fig. S11: The scores 3D plot obtained from PCA (Principle Component Analysis) based on Raman spectra in two different projections for 6 groups of human colon cells: control group (blue square), normal cells treated by tBHP (turquoise square), normal cells treated by tBHP and supplemented with vitamin C in concentration of 5 µM (green square), normal cells treated by tBHP and supplemented with vitamin C in concentration of 25 µM (pink square), normal cells treated by tBHP and supplemented with vitamin C in concentration of 50 µM (violet square) and cancer cells (red square) obtained for cellular substructure -nucleus.

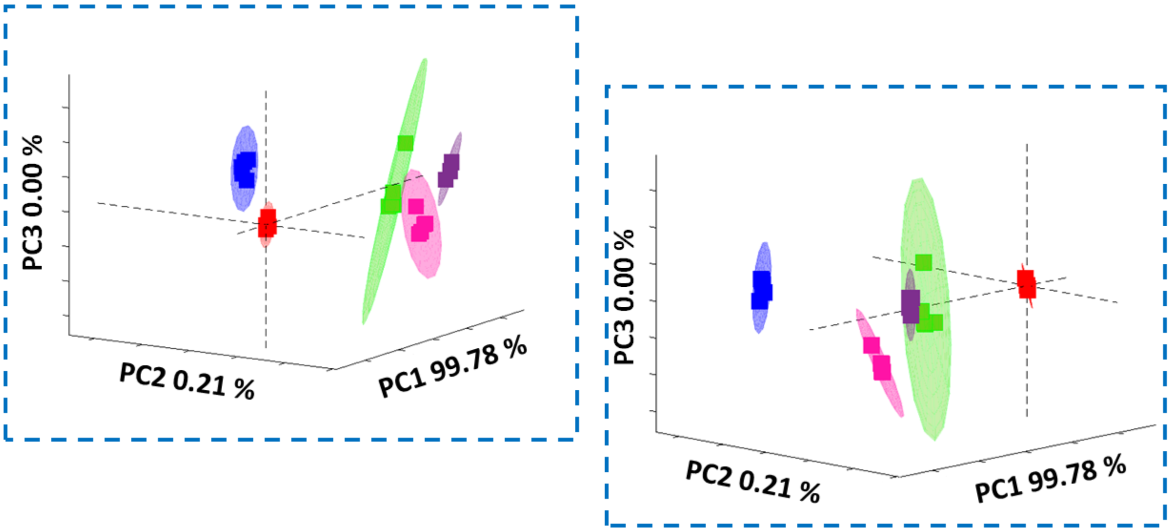

Fig. S12: The scores 3D plot obtained from PCA (Principle Component Analysis) based on Raman spectra in two different projections for 5 groups of human stomach cells: control group (blue square), cancer cells supplemented with vitamin E in concentration of 5 µM (green square), cancer cells supplemented with vitamin E in concentration of 25 µM (pink square), cancer cells supplemented with vitamin E in concentration of 50 µM (violet square) and cancer cells (red square) obtained for cellular substructure -nucleus.

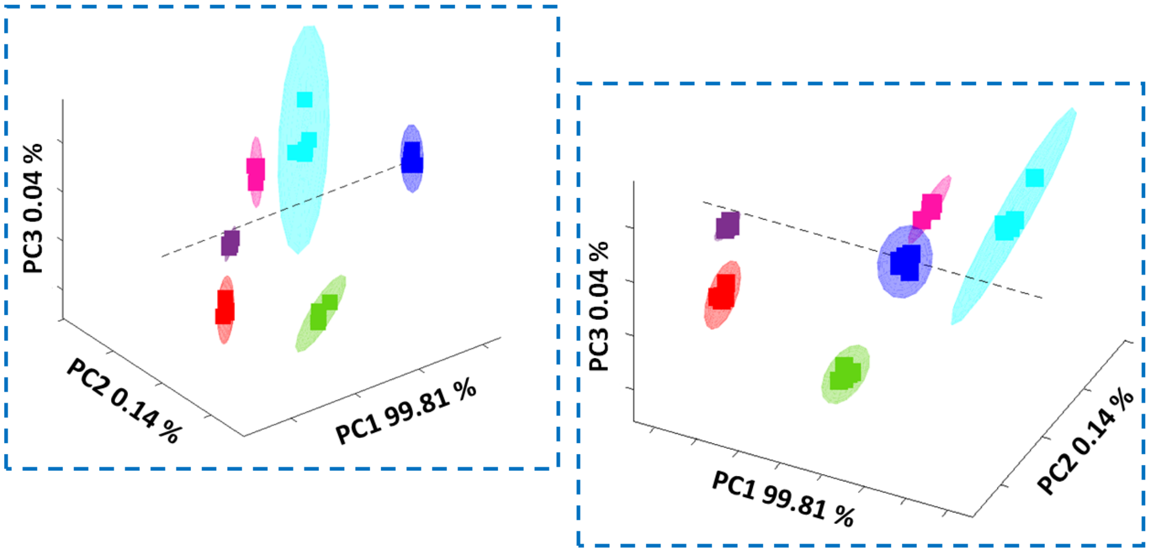

Fig. S13: The scores 3D plot obtained from PCA (Principle Component Analysis) based on Raman spectra in two different projections for 6 groups of human colon cells: control group (blue square), normal cells treated by tBHP (turquoise square), normal cells treated by tBHP and supplemented with vitamin C in concentration of 5 μM (green square), normal cells treated by tBHP and supplemented with vitamin C in concentration of 25 μM (pink square), normal cells treated by tBHP and supplemented with vitamin C in concentration of 50 μM (violet square) and cancer cells (red square) obtained for cellular substructure -mitochondrion.

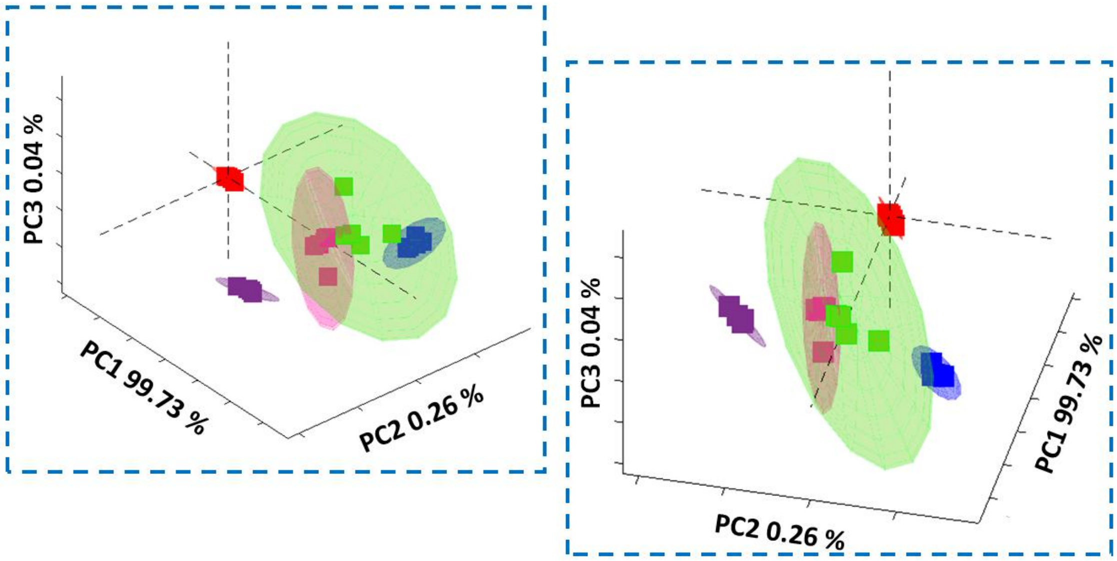

Fig. S14: The scores 3D plot obtained from PCA (Principle Component Analysis) based on Raman spectra in two different projections for 5 groups of human stomach cells: control group (blue square), cancer cells supplemented with vitamin E in concentration of 5 μM (green square), cancer cells supplemented with vitamin E in concentration of 25 μM (pink square), cancer cells supplemented with vitamin E in concentration of 50 μM (violet square) and cancer cells (red square) obtained for cellular substructure -mitochondrion.

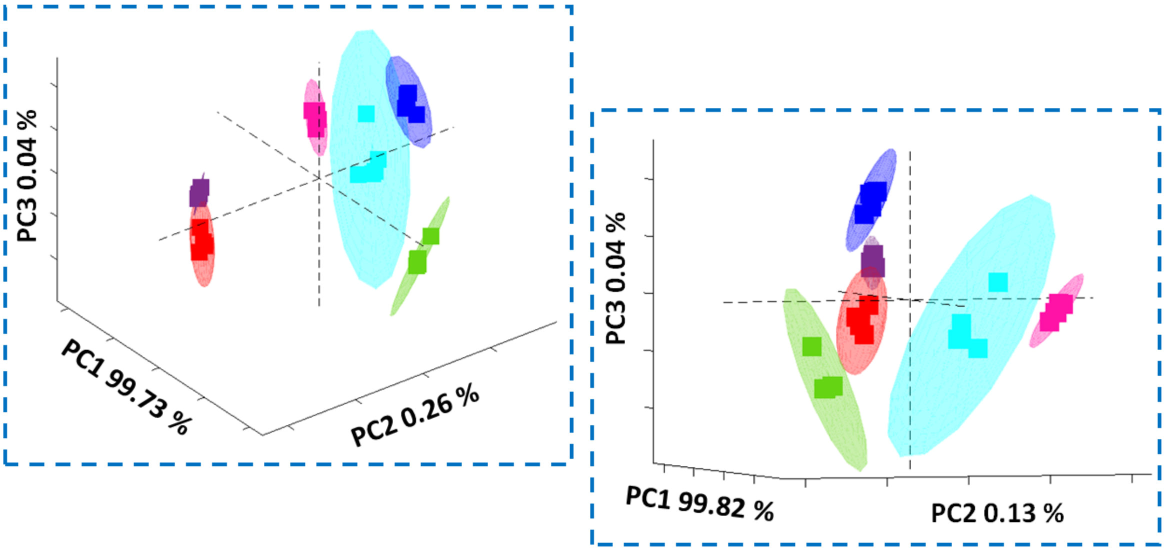

Fig. S15: The scores 3D plot obtained from PCA (Principle Component Analysis) based on Raman spectra in two different projections for 6 groups of human colon cells: control group (blue square), normal cells treated by tBHP (turquoise square), normal cells treated by tBHP and supplemented with vitamin C in concentration of 5 µM (green square), normal cells treated by tBHP and supplemented with vitamin C in concentration of 25 µM (pink square), normal cells treated by tBHP and supplemented with vitamin C in concentration of 50 µM (violet square) and cancer cells (red square) obtained for cellular substructure -lipids - blue region.

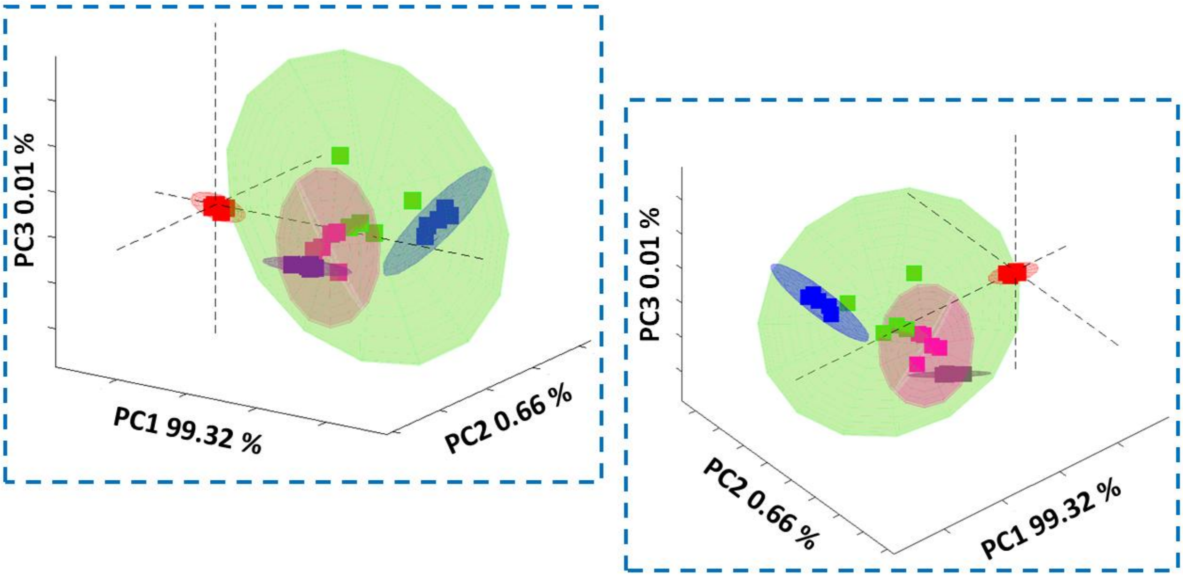

Fig. S16: The scores 3D plot obtained from PCA (Principle Component Analysis) based on Raman spectra in two different projections for 5 groups of human stomach cells: control group (blue square), cancer cells supplemented with vitamin E in concentration of 5 µM (green square), cancer cells supplemented with vitamin E in concentration of 25 µM (pink square), cancer cells supplemented with vitamin E in concentration of 50 µM (violet square) and cancer cells (red square) obtained for cellular substructure - lipids - blue region.

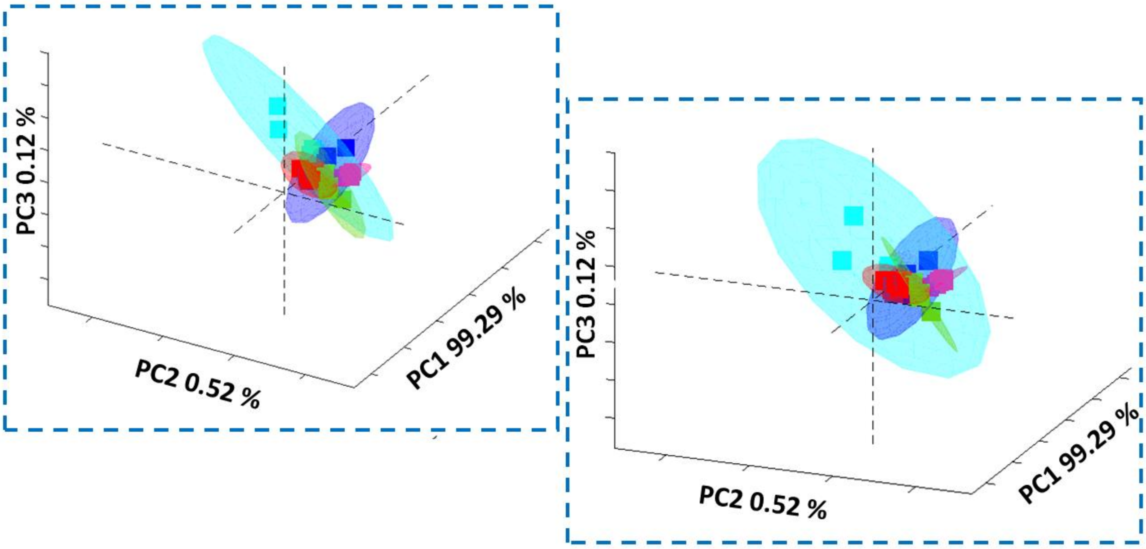

Fig. S17: The scores 3D plot obtained from PCA (Principle Component Analysis) based on Raman spectra in two different projections for 6 groups of human colon cells: control group (blue square), normal cells treated by tBHP (turquoise square), normal cells treated by tBHP and supplemented with vitamin C in concentration of 5 μM (green square), normal cells treated by tBHP and supplemented with vitamin C in concentration of 25 μM (pink square), normal cells treated by tBHP and supplemented with vitamin C in concentration of 50 μM (violet square) and cancer cells (red square) obtained for cellular substructure -lipids - orange region.

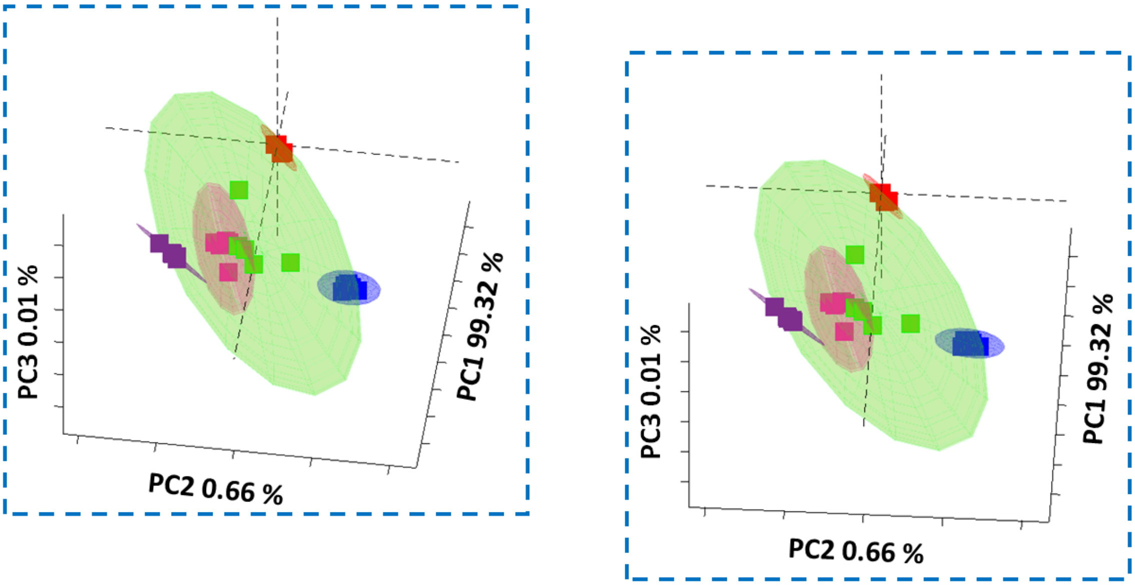

Fig. S18: The scores 3D plot obtained from PCA (Principle Component Analysis) based on Raman spectra in two different projections for 5 groups of human stomach cells: control group (blue square), cancer cells supplemented with vitamin E in concentration of 5 μM (green square), cancer cells supplemented with vitamin E in concentration of 25 μM (pink square), cancer cells supplemented with vitamin E in concentration of 50 μM (violet square) and cancer cells (red square) obtained for cellular substructure -lipids - orange region.

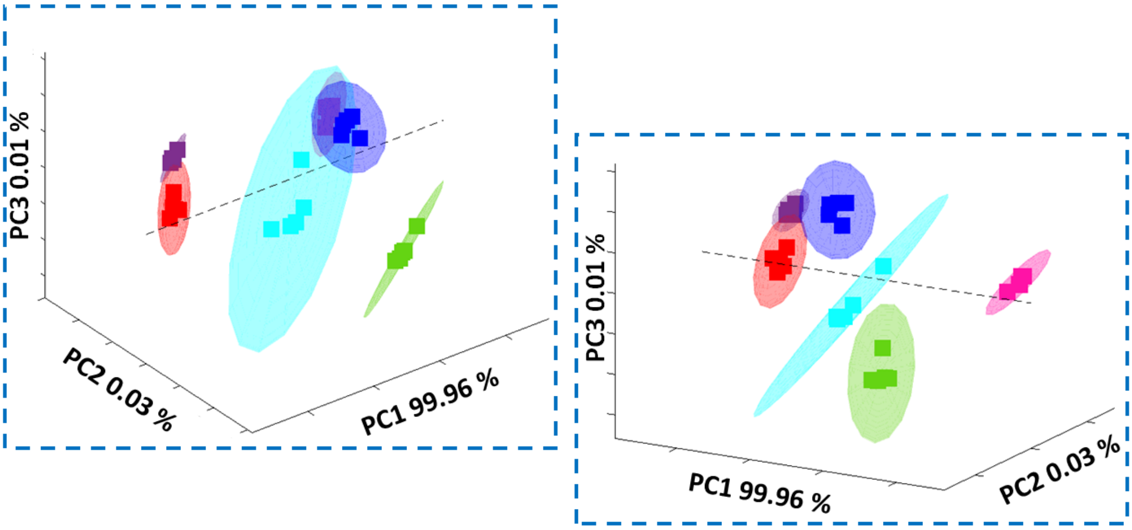

Fig. S19: The scores 3D plot obtained from PCA (Principle Component Analysis) based on Raman spectra in two different projections for 6 groups of human colon cells: control group (blue square), normal cells treated by tBHP (turquoise square), normal cells treated by tBHP and supplemented with vitamin C in concentration of 5 μM (green square), normal cells treated by tBHP and supplemented with vitamin C in concentration of 25 μM (pink square), normal cells treated by tBHP and supplemented with vitamin C in concentration of 50 μM (violet square) and cancer cells (red square) obtained for cellular substructure -cytoplasm.

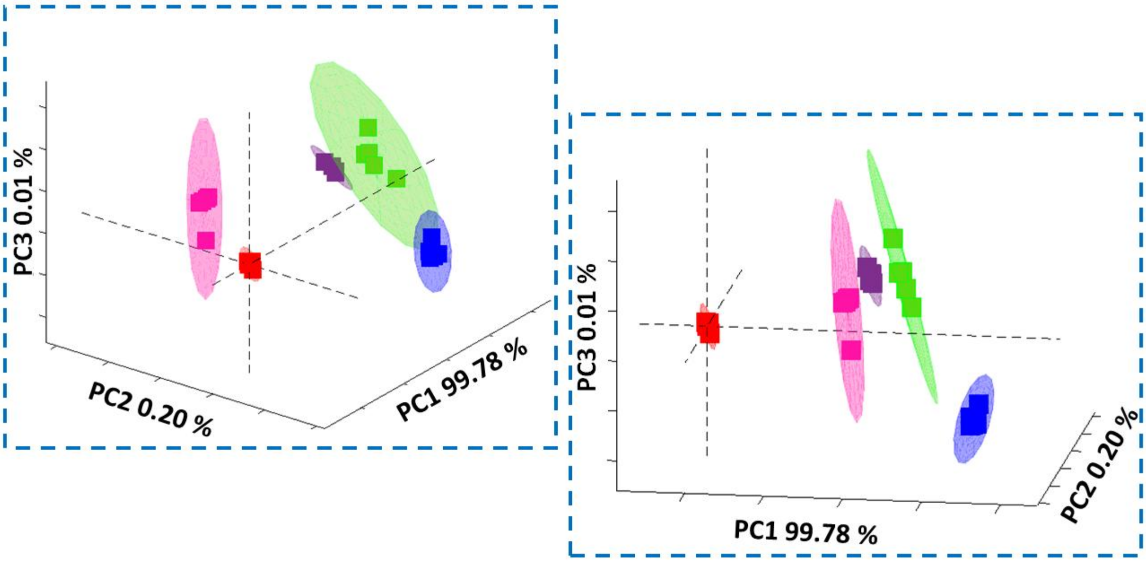

Fig. S20: The scores 3D plot obtained from PCA (Principle Component Analysis) based on Raman spectra in two different projections for 5 groups of human stomach cells: control group (blue square), cancer cells supplemented with vitamin E in concentration of 5 μM (green square), cancer cells supplemented with vitamin E in concentration of 25 μM (pink square), cancer cells supplemented with vitamin E in concentration of 50 μM (violet square) and cancer cells (red square) obtained for cellular substructure - cytoplasm.

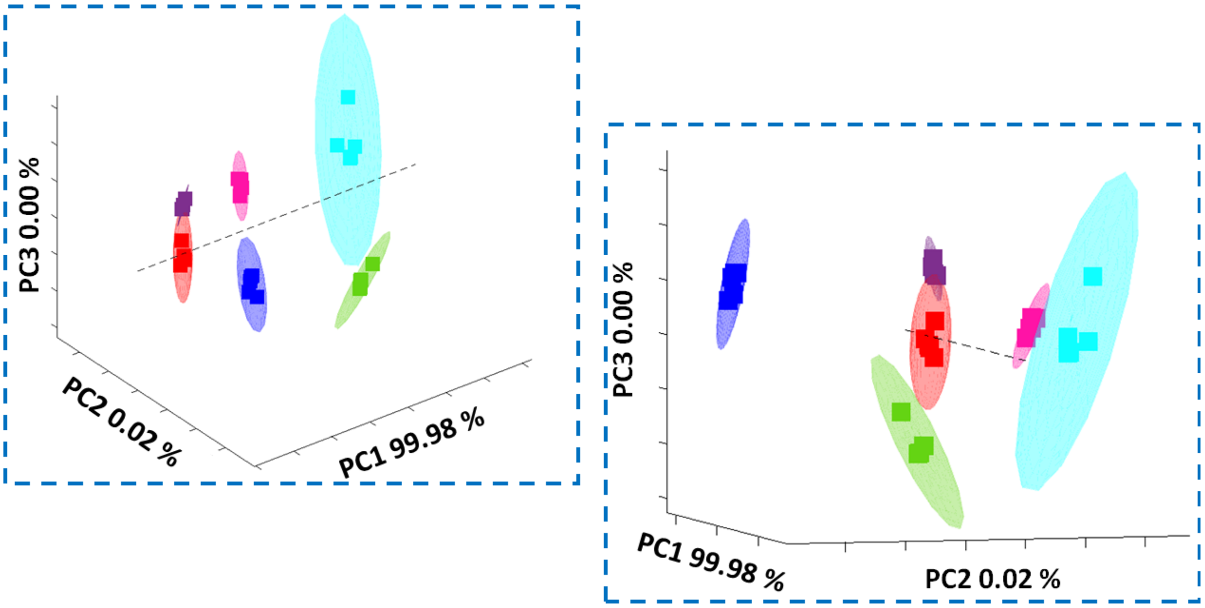

Fig. S21: The scores 3D plot obtained from PCA (Principle Component Analysis) based on Raman spectra in two different projections for 6 groups of human colon cells: control group (blue square), normal cells treated by tBHP (turquoise square), normal cells treated by tBHP and supplemented with vitamin C in concentration of 5 μM (green square), normal cells treated by tBHP and supplemented with vitamin C in concentration of 25 μM (pink square), normal cells treated by tBHP and supplemented with vitamin C in concentration of 50 μM (violet square) and cancer cells (red square) obtained for cellular substructure -membrane.

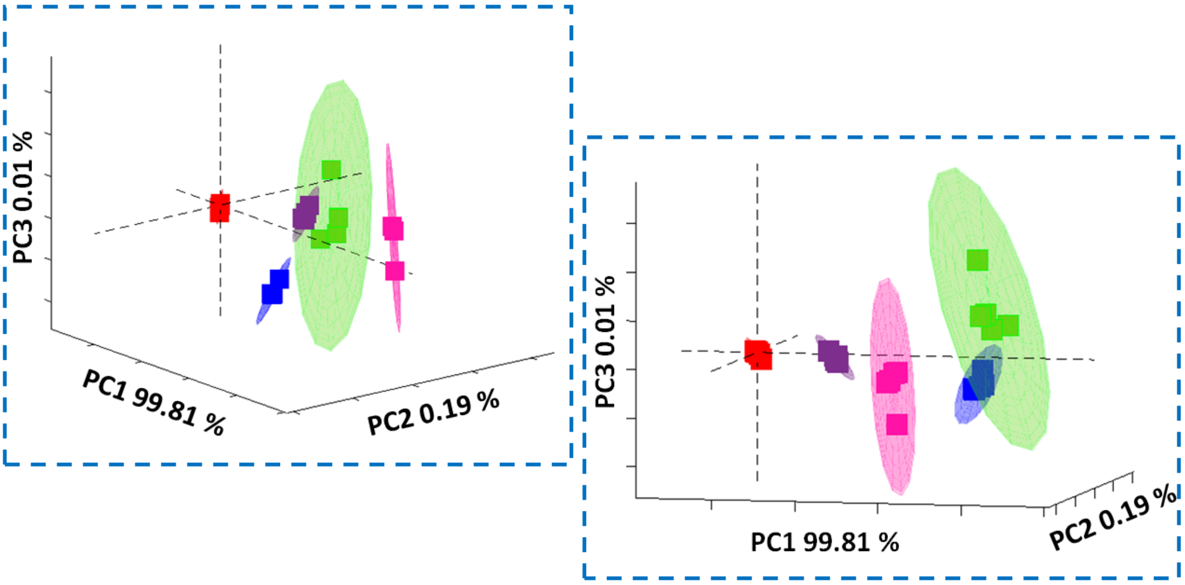

Fig. S22: The scores 3D plot obtained from PCA (Principle Component Analysis) based on Raman spectra in two different projections for 5 groups of human stomach cells: control group (blue square), cancer cells supplemented with vitamin E in concentration of 5 μM (green square), cancer cells supplemented with vitamin E in concentration of 25 μM (pink square), cancer cells supplemented with vitamin E in concentration of 50 μM (violet square) and cancer cells (red square) obtained for cellular substructure -membrane.

To test the classification potential for the investigated colon and stomach cell lines: normal, cancerous and supplemented by antioxidants in the form of vitamins in different concentrations we performed partial least squares discriminant analysis (PLSDA). PLSDA is a chemometric technique widely used to optimize separation between different groups of samples.

Variable importance in projection (VIP) scores greater than 1 help us to identify the spectral regions that are most important in providing optimal PLS-DA model performance. The Raman bands that were most discriminatory in this study are highlighted in bold and can be observed in Figures 23A and 24A and offset in 23B and 24B. Raman bands attributed to lipids (1304, 1444 cm^{-1}), proteins (1258, 1658 cm^{-1}) and nucleic acids (1004, 1078 cm^{-1}) were found to be the most important spectroscopic signatures for discrimination of live cells of human colon and stomach origin and enable discrimination not only of immortalized cancer and fibroblast cells, but also supplemented by different compounds cell samples.

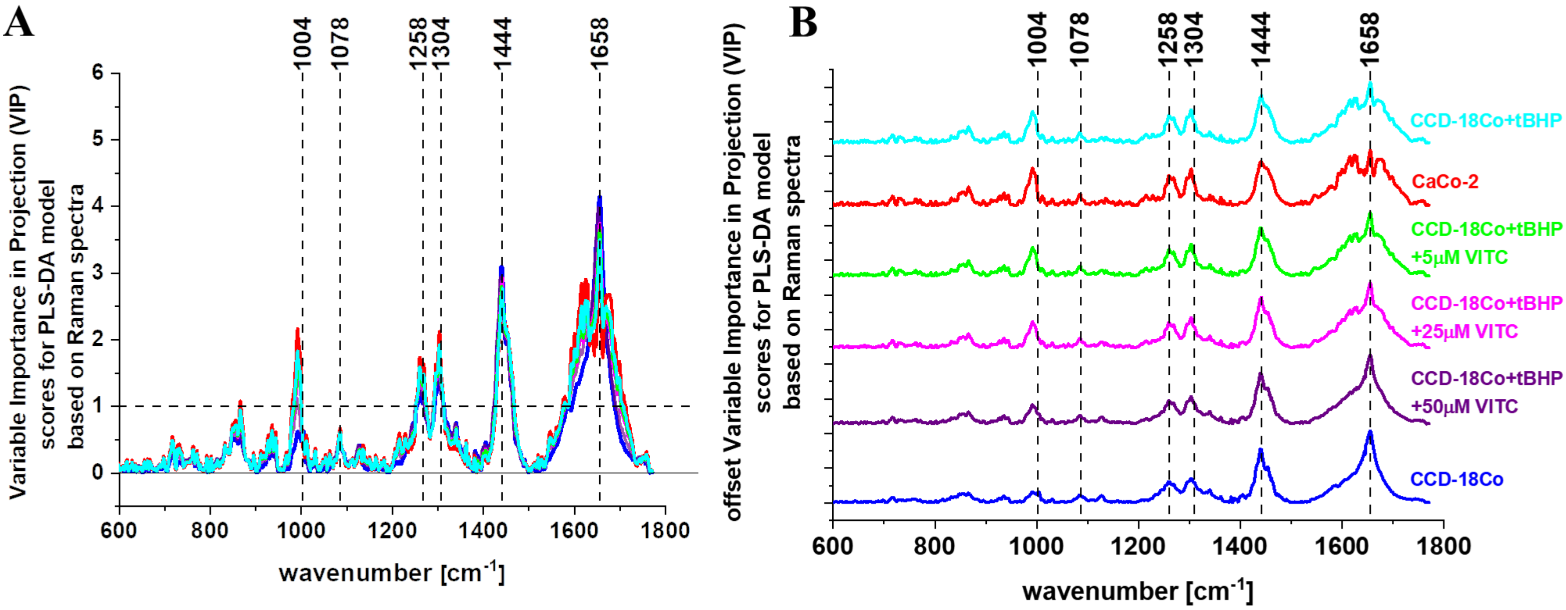

Fig. S23: Variable Importance in Projection (VIP) scores for PLS-DA model based on Raman data for colon cell line: normal, normal supplemented with ROS generating agent (tBHP) and vitamin C in concentration 5, 25, 50 μM and cancerous. (A) VIP scores, shown offset for direct comparison in (B). Raman bands attributed to lipids (1304, 1444cm^{-1}), proteins (1258, 1658 cm^{-1}), nucleic acids (1004, 1078, cm^{-1}) can be observed to be the most important spectroscopic signatures for discrimination of the cell lines.

**Modeled Class**	**Normal**	**Cancer**	**tBHP**	**tBHP + 5 μM**	**tBHP + 25 μM**	**tBHP + 50 μM**
**Sensitivity (Cal):**	1.000	1.000	1.000	1.000	1.000	1.000
**Specificity (Cal):**	0.800	1.000	0.600	0.800	0.800	1.000
**Sensitivity (CV):**	0.667	1.000	0.667	1.000	0.667	1.000
**Specificity (CV):**	1.000	0.867	0.600	0.800	1.000	1.000

Table S1: PLSDA classification quantified according to sensitivity/specificity for colon cell line: normal, normal supplemented with ROS generating agent (tBHP) and vitamin C in concentration 5, 25, 50 μM and cancerous.

The spectra presented as VIP in Figure 23B show very well the effect of the antioxidant vitamin C on healthy cells of the intestine. On the basis of the attached spectra, it can be seen that the most effective

is the highest concentration of vitamin C, which is presented by the spectrum and its shape. As the concentration of vitamin C added to the stressed cells increases, the Raman spectrum gets closer and closer to the seed spectrum. The figure also shows that a lower concentration of vitamin C reduces the effect of oxidative stress, but not as strongly as higher concentrations. On the basis of the spectra, it can also be clearly stated that the spectrum of a cancer cell and a healthy cell subjected to oxidative stress are very similar, which confirms the fact that oxidative stress can lead to the transformation of cells into cancerous forms, with genetic material disturbed as a result of mutations.

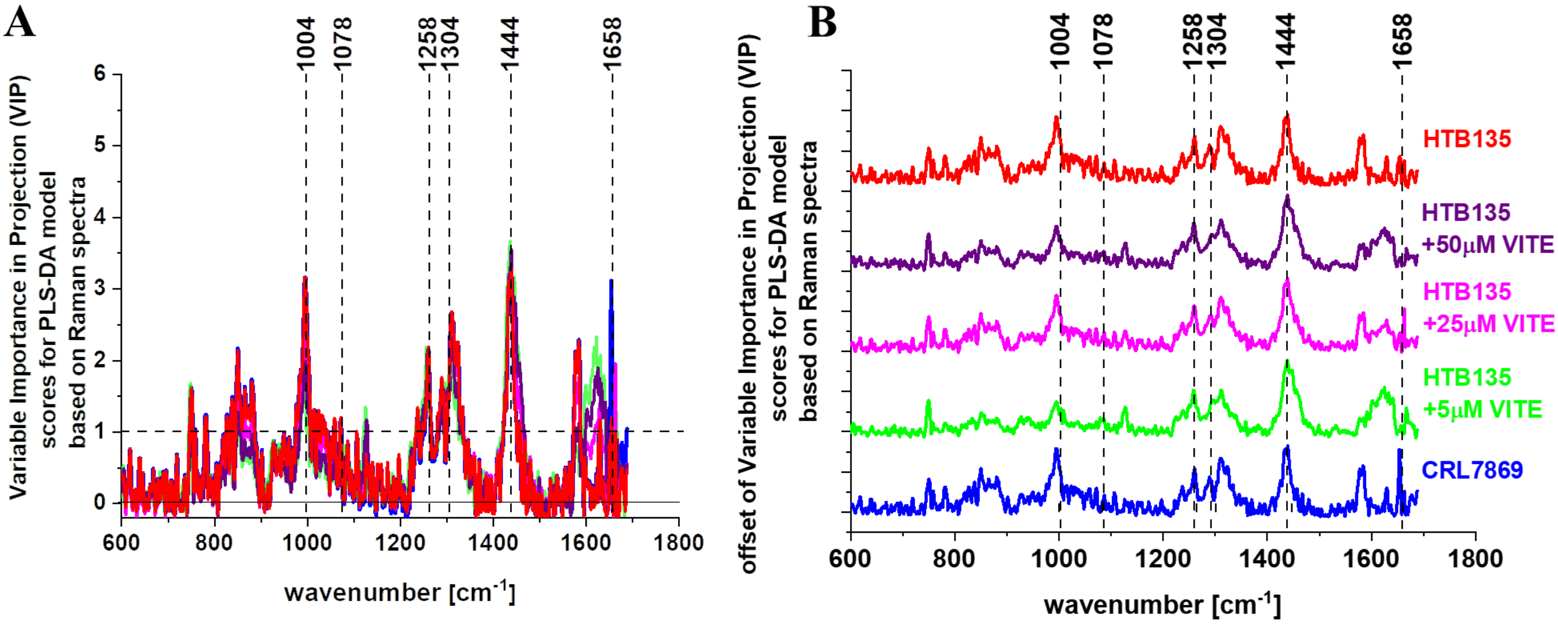

Fig. S24: Variable Importance in Projection (VIP) scores for PLS-DA model based on Raman data obtained for stomach cell line: normal, cancerous and cancerous supplemented with vitamin E in concentration 5, 25, 50 µM. (A) VIP scores, shown offset for direct comparison in (B). Raman bands attributed to lipids (1304, 1444cm^{-1}), proteins (1258, 1658 cm^{-1}), nucleic acids (1004, 1078, cm^{-1}) can be observed to be the most important spectroscopic signatures for discrimination of the cell lines.

**Modeled Class**	**Normal**	**Cancer**	**5 µM**	**25 µM**	**50 µM**
**Sensitivity (Cal):**	1.000	1.000	1.000	1.000	1.000
**Specificity (Cal):**	0.750	0.833	0.500	0.500	0.500
**Sensitivity (CV):**	0.667	0.667	1.000	1.000	1.000
**Specificity (CV):**	0.750	0.833	0.500	0.500	0.500

Table S2: PLSDA classification quantified according to sensitivity/specificity for stomach cell line: normal, cancerous and cancerous supplemented with vitamin E in concentration 5, 25, 50 µM.

All presented results on VIP proved that metabolic regulations in cancer may involve genetic modifications and a change in metabolite pattern, with higher amino acid level, upregulated lipogenesis and upregulation of nucleotide metabolism and synthesis. Moreover, HTB-135 cells exposed to different vitamin E concentrations show further changes in band at 1004, 1078, 1258,

1304, 1444, 1658 cm^{-1} indicating that vitamin E can affect intensity of Raman bands typical for nucleic acids, proteins and lipids in HTB-135 cells.

The effect of vitamin E activity on tumor cells is smaller than that observed for vitamin C. However, it is apparent from the figures above that vitamin E acts as an inhibitor of carcinogenesis in gastric cancer. It is obvious that only the use of vitamin E will not eliminate the cancerogenesis process, while its positive effect on stomach cells is confirmed by the spectra in Figure 24B.

Comparing data acquired from human stomach and colon cancer and fibroblast normal colon and stomach cell lines indicates that the Raman spectral features associated with lipids, proteins and nucleic acids (bands at 1004, 1258, 1304, 1444, 1658 cm^{-1}) prove to be potentially useful biomarkers for monitoring the progression of carcinogenesis, effect of supplementation by using antioxidants in line with previous studies.
